# Tetraparesis following thoracic spine surgery in a patient with Klippel–Feil syndrome and ABCB4 mutation: a case report

**DOI:** 10.1186/s13256-023-04263-8

**Published:** 2023-12-23

**Authors:** Michele Da Broi, Aria Nouri, Gildas Patet, Luca Paun, Andrea Bartoli, Granit Molliqaj, Karl Schaller, Enrico Tessitore

**Affiliations:** 1 Department of Clinical Neurosciences, Division of Neurosurgery, Geneva University Hospitals, Geneva, Switzerland; 2 Division of Neurosurgery, Department of Neuroscience, University of Cambridge, Cambridge, UK; 3 Department of Neurosurgery, Division of Spine Surgery, Hospices Civils de Lyon, Lyon, France; 4 Department of Neurosurgery, GHU Site Sainte-Anne, 75014 Paris, France; 5 Université de Paris, 102-108 Rue de La Santé, 75014 Paris, France; 6 Institut de Psychiatrie et Neurosciences de Paris (IPNP), UMR S1266, INSERM, 75014 Paris, IMA-BRAIN France

**Keywords:** Klippel–Feil syndrome, Congenital cervical fusion, Low phospholipid associated cholelithiasis, ABCB4 mutation, Cervical myelopathy, Case report

## Abstract

**Background:**

Klippel–Feil syndrome is a rare condition described in 1912 by Maurice Klippel and André Feil. It is defined as a congenital cervical fusion of at least two vertebrae, associated with a classical triad of clinical signs: short neck, low posterior hairline, and limited range of movement. However, Klippel–Feil syndrome manifests with a vast spectrum of phenotypes, ranging from no symptoms to complete triad, with or without other associated malformations. Most commonly, CCF results from sporadic mutations, even though autosomal recessive, autosomal dominant, or even X-linked inheritance can be detected. The *ATP-binding cassette subfamily B member 4* is only expressed in the liver and is involved in biliary phospholipid secretion. The clinical spectrum includes various hepatobiliary pathologies, including low phospholipid-associated cholelithiasis, and has never been associated with musculoskeletal anomalies.

**Case presentation:**

A 55-year-old male Caucasian patient presenting with low phospholipid-associated cholelithiasis syndrome with ATP-binding cassette subfamily B member 4 mutation and liver cirrhosis was referred to our clinic for a liver transplant. A period of 6 months before, the patient underwent a T7–T9 posterior fixation for a T8 osteoporotic fracture. Postoperatively, he was tetraparetic, whereas he was neurologically intact before the operation. At admission to our hospital, he was still tetraparetic and presented with clinical signs of cervical myelopathy. Moreover, he suffered a limitation of cervical range of motion in all directions, short neck, and low posterior hairline. Imaging showed multiple cervical and thoracic vertebral bodies fusion, as well as cervical spine stenosis. Based on the available data, we diagnosed a type 3 Klippel–Feil syndrome according to Samartzis’ classification.

**Conclusions:**

The heterogeneity of KFS and the various potential hereditary links that are known indicate that it is important to highlight all potential cases related to known genetic defects. At present, no association between ATP-binding cassette subfamily B member 4 mutation and congenital cervical fusions has been reported. The other important clinical focus of this case is the appearance of spontaneous tetraparesis after thoracic spine surgery. This mechanism remains unclear, but considering different spinal anatomy it might have been due to difficult intubation and patient’s positioning during his previous operation.

## Background

Klippel–Feil syndrome (KFS) is a relatively uncommon condition first described in 1912 by Maurice Klippel and André Feil [1]. According to a recent demographic study, KFS prevalence is between 0.58% and 0.71%, and it appears to be more common in females with a female-to-male ratio of 4:1 [2]. It is defined as a congenital cervical fusion (CCF) of at least two vertebrae, associated with a classical triad of clinical signs, namely short neck, low posterior hairline, and limited range of movement [3]. However, it has been estimated that only 34–74% of patients present with this constellation of findings [4]. Indeed, KFS manifests with a vast spectrum of phenotypes, ranging from asymptomatic cases and patients presenting an incomplete triad, and cases that include a larger array of congenital anomalies. In the symptomatic population, neck pain is one of the most common symptoms [5].

CCF is believed to result from an abnormal somite segmentation process between the 2nd and the 8th weeks of gestation. There exists specific signaling cascades, which are responsible for a successful somite segmentation and an aberration of any of these elements can lead to congenital deformity [6]. Most commonly, CCF results from sporadic mutations; even autosomal recessive, autosomal dominant, or even X-linked inheritance are also described in the literature. At present, several genes have been identified to play a role in spinal development [7]. For instance, *MEOX1* gene is known as the most common autosomal recessive transmission for CCF [8], and mutations in the notch pathway disrupt somite segmentation.

The *ATP-binding cassette subfamily B member 4* (ABCB4), also known as *multidrug resistance protein 3* (MDR3), encoded by *ABCB4*, is involved in biliary phospholipid secretion, protecting hepatobiliary system from deleterious detergent and lithogenic properties of the bile. Well-established phenotypes of *ABCB4* deficit are progressive familial intrahepatic cholestasis type 3, low phospholipid-associated cholelithiasis (LPAC) syndrome, high gamma-glutamyl transferase intrahepatic cholestasis of pregnancy, chronic cholangiopathy, and adult biliary fibrosis/cirrhosis. Moreover, *ABCB4* aberrations may be involved in some cases of drug induced cholestasis, transient neonatal cholestasis, and parenteral nutrition-associated liver disease [9].

However, in current literature there is no report of association between *ABCB4* mutations and KFS, or more generally CCF. In this case report, we describe a patient affected by LPAC syndrome with Child–Pugh type C cirrhosis, who was waiting for liver transplant and presented with a classical triad of KFS and mutation of *ABCB4*.

## Case presentation

A 55-year-old male Caucasian patient affected by LPAC syndrome with Child–Pugh type C liver cirrhosis presented to our hospital to perform a preoperative work-up for liver transplant.

A period of 6 months before, the patient underwent a T7–T9 posterior fixation and decompression at another hospital for a T8 osteoporotic fracture classified as OF5 according to the classification proposed by the German Society for Orthopedics and Trauma [10]. The operation was complicated by a cement leakage in T8 epidural space. Surprisingly, after surgery the patient presented with tetraparesis that was more severe on the left side and associated with an ipsilateral sensitive deficit. Notably, the patient was neurologically intact before the intervention and the cement leakage did not explain the postoperative neurological deficit of the upper limbs. For this reason, he was admitted to a neuro-reeducation clinic for 5 months and thereafter, he was transferred to our hospital to undergo a liver transplantation. At admission, he was still tetraparetic and due to the persistence of neurological deficit, our colleagues sought an expert neurosurgical consultation.

On physical examination, the patient was tetraparetic and stable compared with the immediate postoperative neurological status described in the clinical documentation. He also showed clinical signs of cervical myelopathy (bilaterally positive Hoffman’s sign, four limbs with symmetrical hyperreflexia, and bilateral ankle clonus). Moreover, he suffered a limitation of cervical motion range in all directions and showed a short neck and low posterior hairline, namely the classical clinical triad of KFS.

**Fig. 1:**
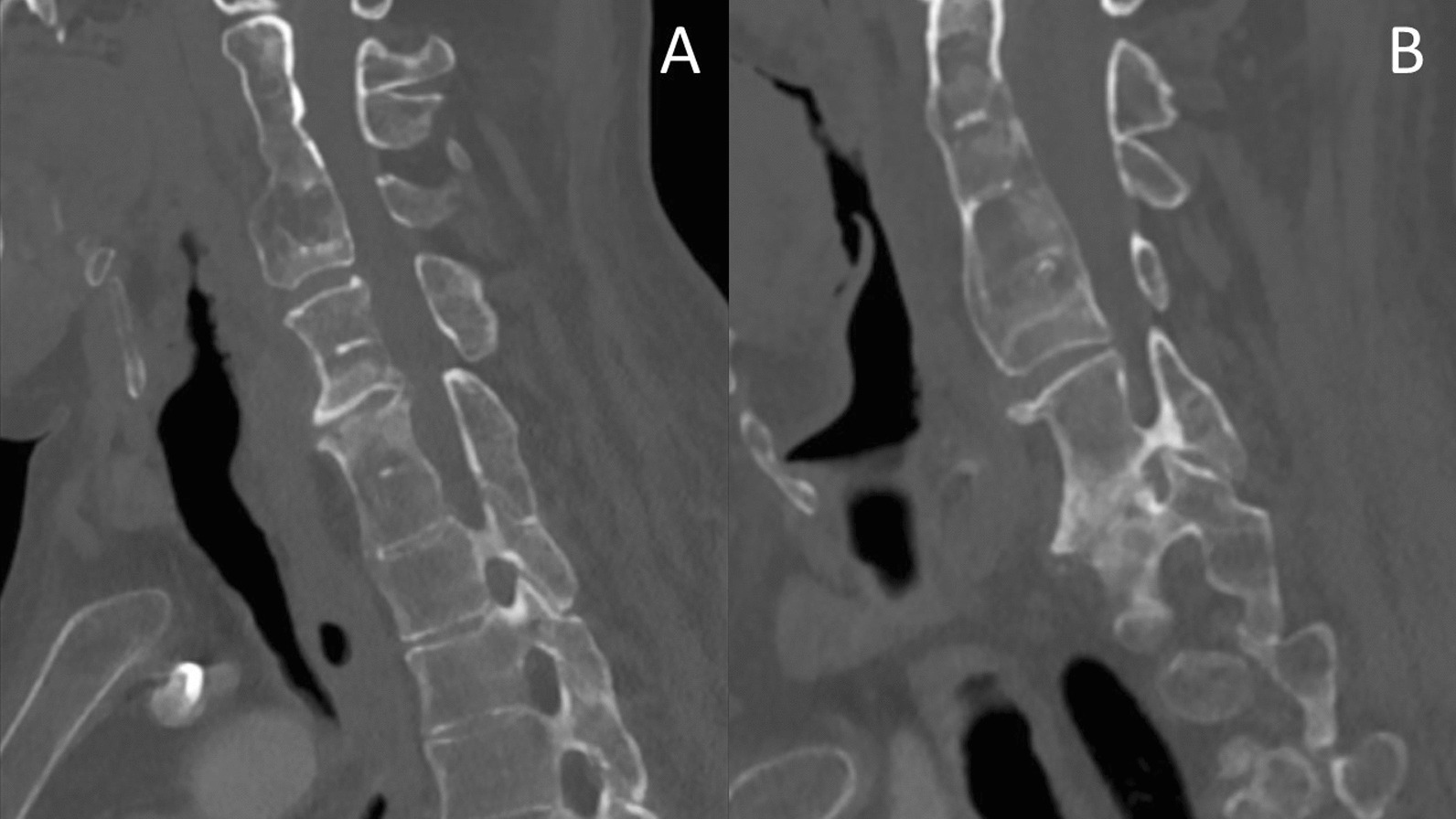
Cervical spine computed tomography scan showing congenital cervical fusion and cervical stenosis. **A** sagittal view showing congenital fusion at C5-C6 and C7-T2 levels. **B** sagittal view showing congenital fusion at C2-C4 levels

**Fig. 2:**
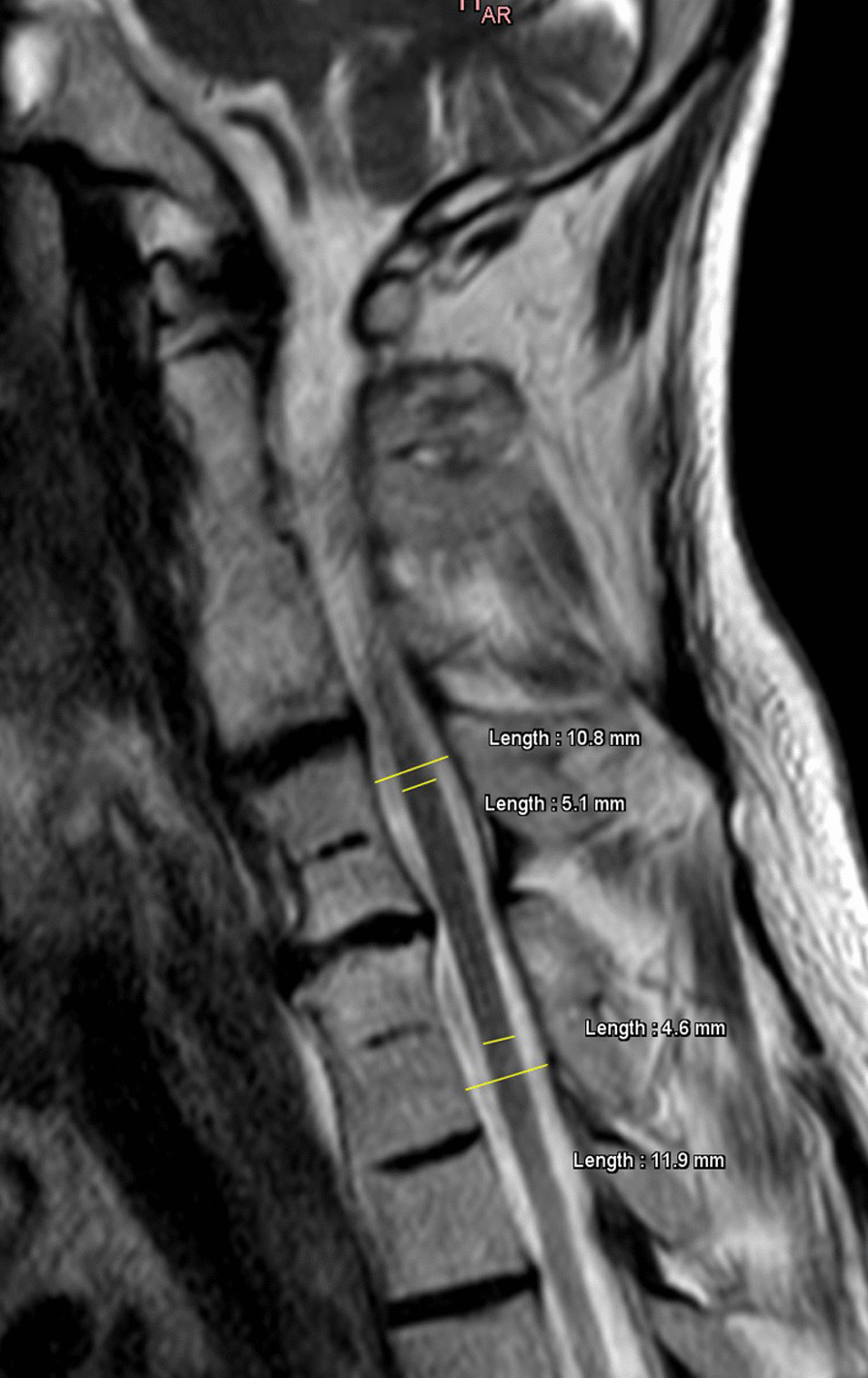
Cervical spine T2-sequence magnetic resonance imaging showing the calculation of spinal cord occupation ratio index. The spinal cord and canal diameters are measured immediately above and below the area of degenerative pathology

**Fig. 3:**
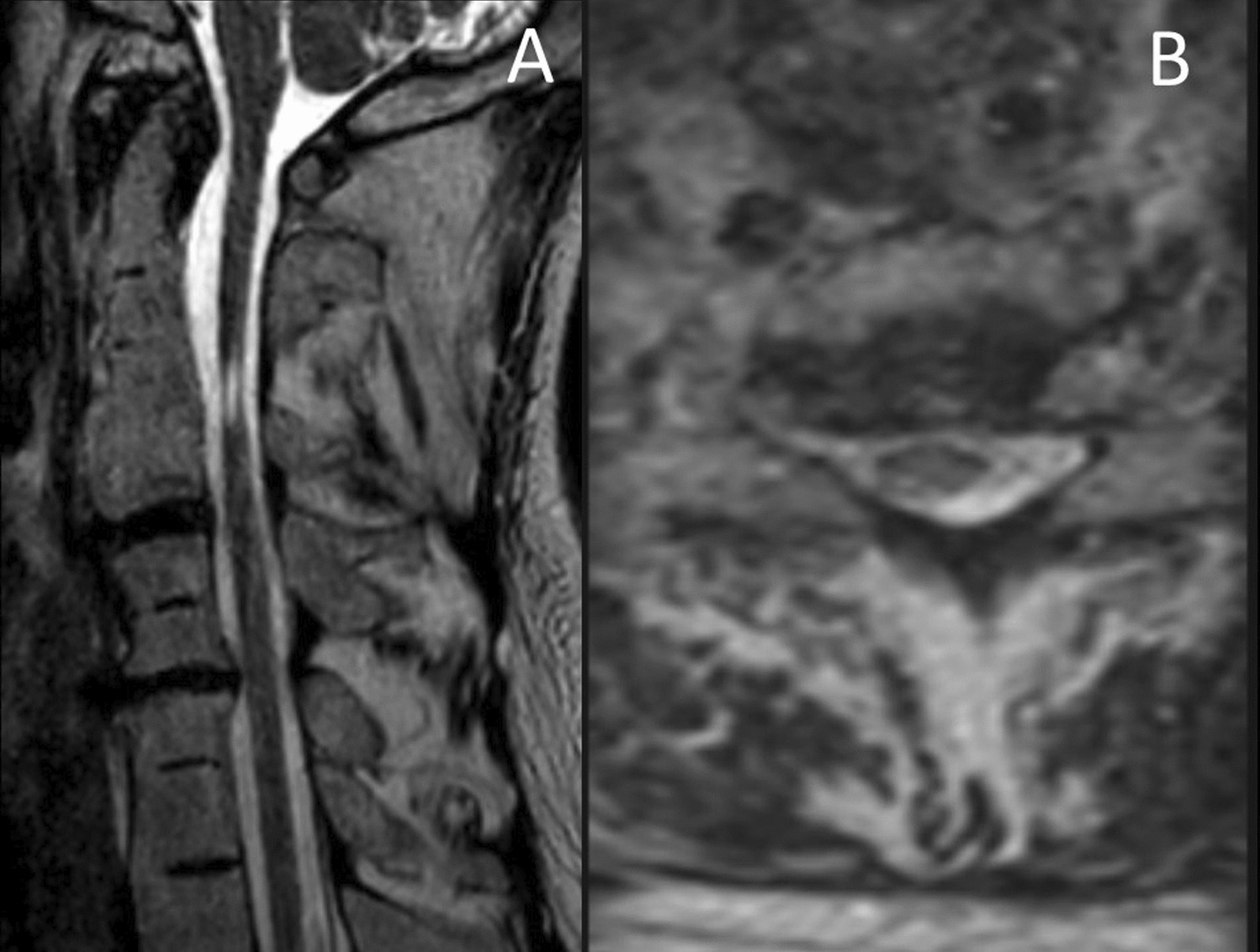
Cervical spine T2-sequence magnetic resonance imaging. **A** sagittal view showing the canal stenosis at C6–C7, as well as the hypersignal at the level of C3. **B** axial view of the C6–C7 level which shows a moderately stenotic cervical canal

**Fig. 4:**
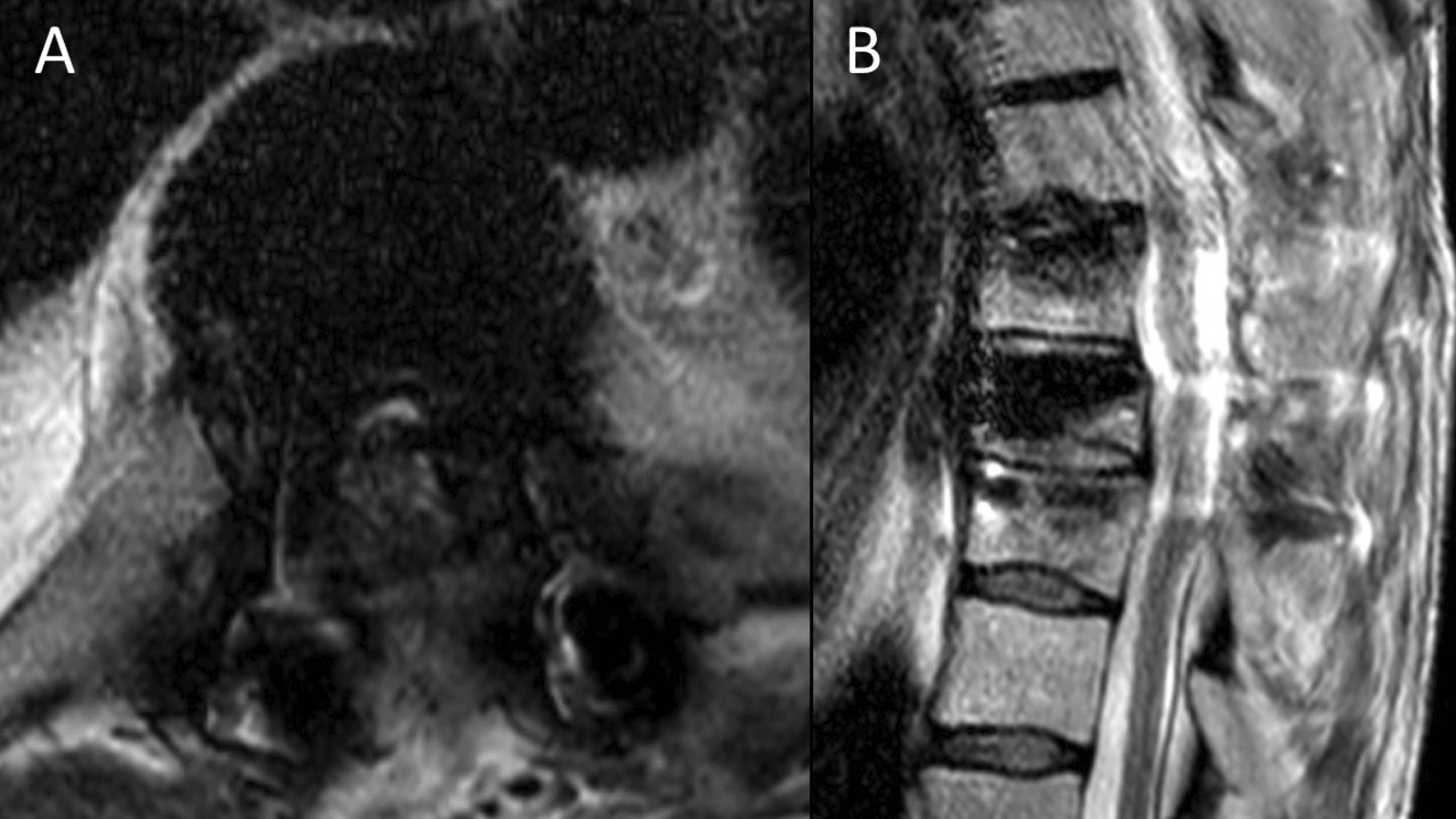
Dorsal spine T2-sequence magnetic resonance imaging showing the kyphoplasty at T8 level with posterior fixation at T7 and T9. **A** axial view showing intracanal cement leakage at T8 level, without visible compressive consequences on the dorsal spinal cord. **B** sagittal view showing no myelopathic consequences at T7-T9 level

Imaging performed at our hospital, showed the fusion of multiple cervical and thoracic vertebral bodies, namely in C2–C4, C5–C6, and C7–T2 [Fig. 1], without congenital cervical spine stenosis spinal cord occupation ratio (SCOR) index of 43% (Fig. 2) (11). T2 sequences underlined a spinal cord hypersignal potentially due to prior injury at the level of the C3 body; however, it was impossible to clearly determine its origin due to the lack of comparative imaging (Fig. 3). At the level of T7–T9 thoracic fixation, no stenotic consequences on the spinal cord due to the cement leakage were found (Fig. 4). According to radiological presentation, a diagnosis of type 3 KFS was made [4]. Even though a T2 hypersignal was detected at C3 level, there was no cervical stenosis at this segment, and it was considered as an ischemic sequalae. Based on these assumptions, an acute spinal cord compression at stenotic levels was unlikely. Since no compression of the spinal cord at T8 was identified and the tetraparesis is not compatible with a spinal cord lesion at T8, our hypothesis was that surgical installation and/or intubation before surgery for T8 fracture might have produced a cervical spinal cord injury (SCI) and consequently produced the neurological deficit.

Based on these considerations, the absence of instability, and stability of neurological findings over the last few months, no surgical treatment was undertaken. Instead, we proposed a radiological follow-up concerning the C6–C7 discopathy and cervical canal stenosis.

## Discussion and conclusions

The main objective of this article is to present a case of syndromic KFS associated to an *ABCB4* genetic mutation. To the best of our knowledge, this case represents the first patient with KFS and *ABCB4* mutation. While we cannot determine if the presentation of this genetic mutation and KFS are related, the KFS heterogeneity and the various potential hereditary links that are known, indicate that it is important to highlight all potential cases related to known genetic defects. For example, it is known that congenital fusion of vertebrae can occur in many different genetic syndromes, such as mucupolysaccaride disease and vertebral defects, anal atresia, cardiac defects, tracheo-esophageal fistula, renal anomalies, and limb abnormalities (VACTERL) syndrome, many of which have been pointed out by Giampietro *et al.* [12].

Genetically, our patient presented with an *ABCB4* mutation, which is involved in biliary phospholipid secretion. This gene is located on chromosome 7q21.1 and it is exclusively expressed in the liver. *ABCB4/MDR3* is organized as a full transporter and acts as an energy-dependent “floppase,” translocating phospholipids of the phosphatidylcholine family from the inner to the outer leaflet of the lipid bilayer of the canalicular membrane to be extracted by bile salts [8]. The main clinical spectrum of *ABCB4* deficiency-associated diseases includes various hepatobiliary pathologies. However, as far as we know, no association is reported between *ABCB4* mutation and CCF or other musculoskeletal problems, which might indicate that this is only a rare coincidence. Moreover, the absence of a family history of spine deformities indicates that KFS in this patient was perhaps a sporadic mutation.

The second important clinical focus of this case was the appearance of spontaneous tetraparesis. The manifestation of this matter remains unclear, but it is possible that it occurred due to a difficult intubation and patient positioning during his previous operation. Unfortunately, the diagnosis of KFS was not known previously, which is often the case, and therefore makes surgical preparation more challenging. Indeed, there exists literature showing how short neck and body habitus in KFS make intubation and positioning more difficult compared with the general population [13, 14]. On the other hand, since the SCI occurred behind what appeared to be a fused segment, it is virtually impossible to outline a clear connection between patient positioning and the development of SCI during the intervention.

## Data Availability

Data sharing is not applicable to this article as no datasets were generated or analyzed during the current study.

## Abbreviations

ABCB4: ATP-binding cassette subfamily B member 4
CCF: Congenital cervical fusions
KFS: Klippel–Feil syndrome
LPAC: Low phospholipid-associated cholelithiasis
MDR3: Multidrug resistance protein 3
MRI: Magnetic resonance imaging
OF: Osteoporotic fracture
SCI: Spinal cord injury
